# An Update on the Current State of SARS-CoV-2 Mac1 Inhibitors

**DOI:** 10.3390/pathogens12101221

**Published:** 2023-10-07

**Authors:** Joseph J. O’Connor, Dana Ferraris, Anthony R. Fehr

**Affiliations:** 1 Department of Molecular Biosciences, University of Kansas, Lawrence, KS 66045, USA; joseph.oconnor@ku.edu; 2 Department of Chemistry, McDaniel College, 2 College Hill, Westminster, MD 21157, USA; dferraris@mcdaniel.edu

**Keywords:** coronavirus, SARS-CoV-2, macrodomain, Mac1, ADP-ribosylation, inhibitors, ADP-ribosylhydrolase

## Abstract

Non-structural protein 3 (nsp3) from all coronaviruses (CoVs) contains a conserved macrodomain, known as Mac1, that has been proposed as a potential therapeutic target for CoVs due to its critical role in viral pathogenesis. Mac1 is an ADP-ribose binding protein and ADP-ribosylhydrolase that promotes replication and blocks IFN responses, though the precise mechanisms it uses to carry out these functions remain unknown. Over the past 3 years following the onset of COVID-19, several groups have used high-throughput screening with multiple assays and chemical modifications to create unique chemical inhibitors of the SARS-CoV-2 Mac1 protein. Here, we summarize the current efforts to identify selective and potent inhibitors of SARS-CoV-2 Mac1.

## 1. Introduction

The advent of COVID-19 highlighted both the importance of rapid responses to viral outbreaks and the necessity of a diverse toolset in implementing those strategies. Though the historic rollout of the COVID-19 vaccines was instrumental to global recovery, there remains a need for ready-made, fast-acting antivirals that can be rapidly deployed when needed. While a vaccine’s development timeline may begin in the early stages of a future pandemic, a plethora of drug-based solutions may be developed in the interim. One promising antiviral drug target is the highly conserved coronavirus macrodomain, known as Mac1.

Macrodomains are small globular protein domains that display a mixed α-β-α sandwich fold structure that is highly conserved across all forms of life [1]. Previous studies have established ADP-ribose as their target ligand [2,3]. ADP-ribosylation is the modification of proteins and nucleic acids via the addition of ADP-ribose subunits by ADP-ribosyltransferases (ARTs) [4]. This occurs with either single subunits or polymers of ADP-ribose, known as mono-ADP-ribosylation (MARylation) or poly-ADP-ribosylation (PARylation). ADP-ribosylation regulates intracellular processes, including DNA damage repair, protein degradation, stress responses, and immune signaling [4]. Furthermore, macrodomains are found in several families of positive-sense RNA viruses and one family of DNA viruses and promote their replication and pathogenesis [5].

SARS-CoV-2 Mac1 is contained within non-structural protein 3 (nsp3) and belongs to the MacroD-type class, characterized via hydrolysis activity against MARylation [6] and also includes human macrodomains MacroD1, MacroD2, PARG, and the PARP9 and PARP14 N-terminal macrodomains. It shares three major regions of homology with related coronaviruses, namely, NAAN and GGG motifs in loop 1 between β3 and α2, a GIF motif in loop 2 between β6 and α5, and a VGP motif between β5 and α4 [7]. Previous biochemical characterizations of ligand binding within the cleft have implicated several stabilizing interactions with ADP-ribose. A conserved interaction between the D22 residue and the N6 of the adenine is critical for substrate binding, while hydrogen bonds between N40 and distal ribose are conserved in catalytically active macrodomains [7,8]. Comparisons between the SARS-CoV-2 Mac1 and human MacroD-type macrodomains reveal a sequence similarity at or below 38%, suggesting a potential for the specific inhibition of viral macrodomains [7], though it should be noted that human macrodomains also encode the previously highlighted structural motifs.

Mac1 is a critical factor for the replication and/or pathogenesis of all CoVs where it has been tested, including MHV, MERS-CoV, and both SARS-CoV and SARS-CoV-2 [9,10,11]. Point mutations in the Mac1-binding pocket sensitize CoVs to ART activity and IFN signaling [10,12]. More recently, we demonstrated that a complete deletion of Mac1 in MHV and MERS-CoV was not recoverable. However, a Mac1 deletion in SARS-CoV-2 was easily recovered, indicating that it is not essential for replication. However, the Mac1 deletion virus was highly sensitive to IFN-γ signaling and replicated poorly and caused no disease in mice [11]. Recombinant alphaviruses and HEV with macrodomain mutations also result in severe defects in virus replication, indicating that macrodomains clearly play important roles during infection. However, the precise function of viral macrodomains in the viral lifecycle remains unclear, though mutational analysis indicates that it targets multiple stages of the lifecycle [13,14,15]. Together, these studies comprise a framework that has established viral macrodomains as druggable targets. Mac1 inhibitors could be used as probes to better understand the function of Mac1 during infection or could be used as antiviral therapeutics. Here, we will comprehensively review the collective efforts among multiple groups to identify and develop SARS-CoV-2 Mac1 inhibitors with the aim of highlighting emerging techniques and chemotypes.

## 2. Virtual and Crystallography Screens Identify Several Mac1 Interacting Compounds

**Figure 1:**
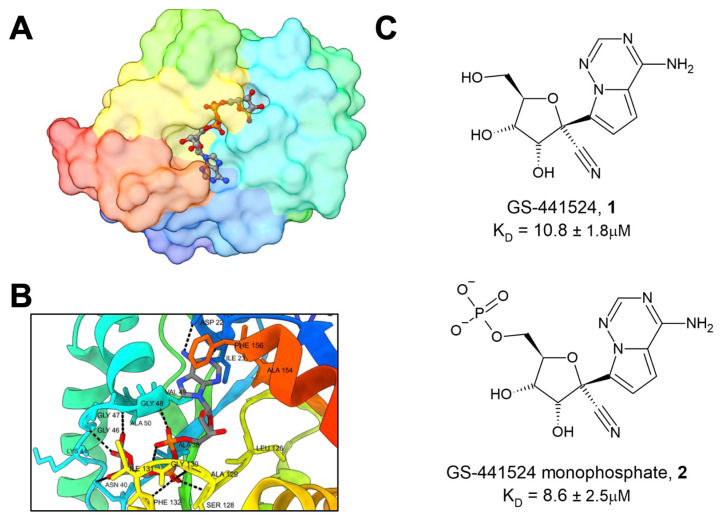
The structure of SARS-CoV-2 Mac1. (**A**) SARS-CoV-2 Mac1 complexed with ADP-ribose within the active site (PDB 6WOJ). (**B**) Coordination of ADP-ribose with the binding pocket of Mac1 is reliant on a network of coordinated hydrogen bonds, critically with residues D22, N40, and a GIF motif at 130–132. (**C**) GS-441524, the active metabolite of remdesivir, with its phosphonated version.

Several groups initially focused on determining the structure and biochemical activities of Mac1 and related macrodomains [1,6,8,16]. Dozens of different Mac1 structures in complex with a variety of ligands are available in the PDB. These structures have included not only the main substrate of Mac1 and ADP-ribose (Figure 1A,B), but also several nucleotides and compounds, including HEPES, MES, GMP, cAMP, and ADP-ribose-2”-phosphate [17,18]. These structural analyses have revealed three pockets near the active site of the enzyme. The two pockets that comprise the adenosine binding site have the highest solvent exposure and together are the preeminent site of interest for ligand design. One especially interesting ligand that has been crystallized with Mac1 is GS441524 (**1**), the active metabolite of remdesivir, an approved anti-CoV drug that is well known for its ability to inhibit CoV polymerase [18]. Compound **1** (K_D_ = 10.8 ± 1.8 μM) and the phosphonated version, **2**, demonstrated low micromolar binding affinity against Mac1 (K_D_ = 8.6 ± 2.5 μM) (Figure 1C). Tsika et al. found that, when used in 500-fold excess, **1** can inhibit Mac1 ADP-ribosylhydrolase activity, and this activity was specific for the SARS-CoV-2 Mac1, as other viral or human macrodomains were not similarly affected [19]. However, isothermal titration calorimetry (ITC) data demonstrated that the compound bound to SARS- and MERS-Mac1 proteins, but with reduced affinity. These results raise the possibility that remdesivir’s Mac1 inhibitory activity may play a small role in its antiviral activity against SARS-CoV-2. However, this hypothesis has not been formally tested. Also, due to its inhibitory activity against the CoV polymerase, remdesivir would not make for a useful Mac1-specific inhibitor, though it may indicate a potential role as a dual polymerase/Mac1 inhibitor.

Concurrent with these structural efforts, several groups have performed virtual screens and biochemical binding assays to identify potential Mac1 inhibitors. Babar et al., Pandey et al., Singh et al., Selvaraj et al., and Russo et al. each used virtual screening to identify several compounds that had docking scores as good or better than ADP-ribose [20,21,22,23]. However, none of these compounds have yet to be confirmed to interact or inhibit Mac1 in biochemical assays. Russo et al. tested 69 different virtual screen hits in a cell-based de-MARylation assay, but none of them demonstrated any inhibition of enzyme activity.

**Figure 2:**
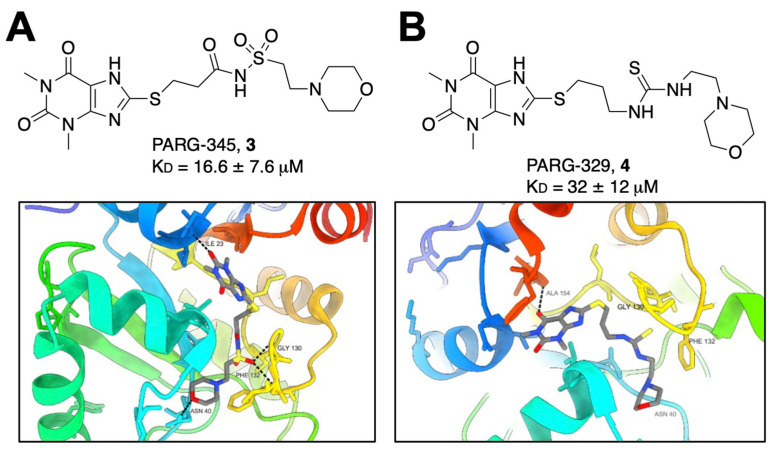
Morpholine-based compounds PARG-345 and PARG-329 bind the Mac1 active site. (**A**) Crystallography shows that PARG-345 (PBD 7LG7) makes several hydrogen bonds within the Mac1-binding pocket, with the methylxanthine group binding at the adenine binding site. (**B**) PARG-329 (PDB 7KXB) binds in the same orientation; however, it interacts with Mac1 primarily through water-mediated contacts (waters not shown for clarity) and takes on a more strained conformation [24].

Knowing that poly(ADP-ribose) glycohydrolases (PARGs) are structurally related to Mac1, and that there are several PARG inhibitors, Brosey et al. utilized virtual screening and scaffold optimization to identify PARG-inhibitor-related morpholine-based structures that might also bind to Mac1 [24]. From this work, the group discovered two compounds, PARG-345 (**3**) and PARG-329 (**4**), that interacted with Mac1 via crystallography. Both compounds anchor the methylxanthine head in the adenine binding site. The morpholine group on PARG-345 interacts with N40 while the sulfonyl linker makes backbone contacts with the G130 and F132 residues of Loop 2 (Figure 2A). Conversely, the thio-urea of PARG-329 interacts with Mac1 largely through water-mediated contacts. The morpholine group extends further into the active site but loses the hydrogen bonding with N40 (Figure 2B). While these compounds interact with Mac1, demonstrating K_D_ values of between 16.6 and 32 μM, no further reports have been published demonstrating any inhibitory activity of these compounds.

**Figure 3:**
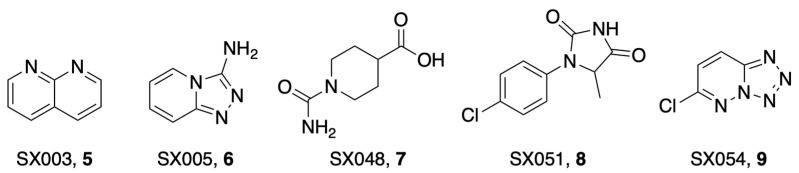
Small binding fragments were identified using SpotXplorer0-generated pharmacophore sets that were mapped to Mac1 via the FTMap algorithm. All interact with the F156 side chain through π-π stacking [25].

To streamline fragment screens, Bajusz et al. developed a new strategy of assembling fragment libraries that utilize confirmed binding pharmacophores [25]. Their pilot library, termed SpotXplorer0, utilized ~3000 experimental protein–fragment structures from the PDB which were then used to generate pharmacophore sets via the FTMap protein-mapping algorithm. The validated workflow, when applied to the SARS-CoV-2 Mac1, identified five small fragments that crystallized with Mac1: SX003, SX005, SX048, SX051, and SX054 (**5**–**9**) (Figure 3). Compounds **5**, **6**, and **9** bind in the adenine binding site of Mac1 and all target the aromatic sidechain of F156, utilizing π-π stacking interactions, while **7** and **8** occupy the proximal ribose site. None of these molecules were tested for Mac1 inhibition, but they could offer viable starting points for further compound iteration.

**Figure 4:**
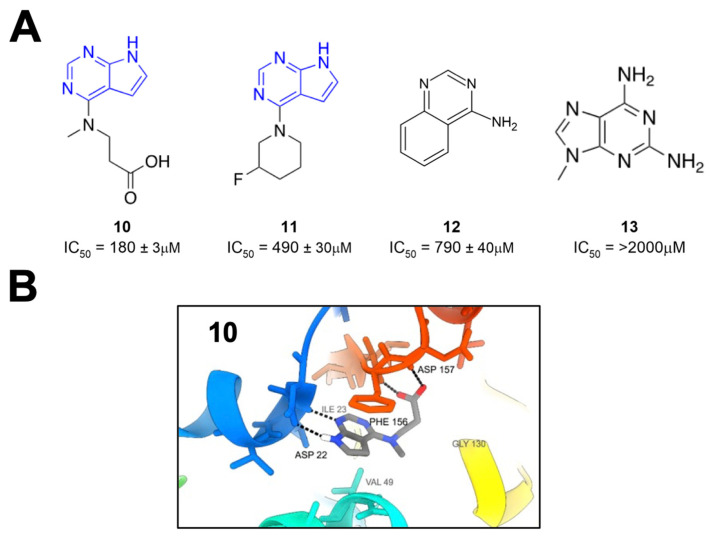
A large crystallography screen identifies several small-molecule Mac1 binders. (**A**) Fragments identified from crystallography screen for Mac1 binders. (**B**) A 7H-pyrrolo[2,3-d]pyrimidine moiety anchors fragment **10** within the adenine binding site (PDB 5RSG) [26].

Schuller et al. took crystallographic screening to the next level by performing a crystallography screen of >2500 diverse fragments, which identified 214 Mac1-binding fragments [26]. In addition, they also used computational docking of 20 million fragments, from which they tested 60 fragments and confirmed 20, for a total of 234 confirmed Mac1 binders. Of these hits, about 80% occupied the active site of the enzyme, while the remaining hits were scattered across the surface of the protein and in a conserved pocket near K90. The most common and effective structure amongst these fragments was a 7H-pyrrolo[2,3-d]pyrimidine, a component of compounds **10** and **11** (Figure 4), which use hydrogen bonds with D22, I23, and π interactions with F156 to anchor fragments in the adenine-binding pocket. Many compounds also included carboxylic acid (e.g., **10**), which formed hydrogen bonds with the backbone amides of F156 and D157 in the “oxyanion” subsite (Figure 4). Combined with Bajusz et al.’s screen, a notable hallmark of these fragment screens was the identification of fused heterocyclic rings that anchor the fragments to D22, I23, and/or F156. In addition to crystallography, several fragments also showed a thermal shift with Mac1 in a differential scanning fluorimetry (DSF) assay, had measurable binding via ITC, and showed the inhibition of Mac1 activity in a peptide displacement assay using a homogenous time-resolved fluorescence assay (HTRF) similar to the previously developed AlphaScreen assay (**10**–**13**) [27]. The IC_50_ values for inhibition ranged from 180 μM (**10**) to more than 2 mM (**13**), which are reasonable values for fragments. These fragments would prove to serve as excellent starting points for further inhibitor derivatization (see below).

**Figure 5:**
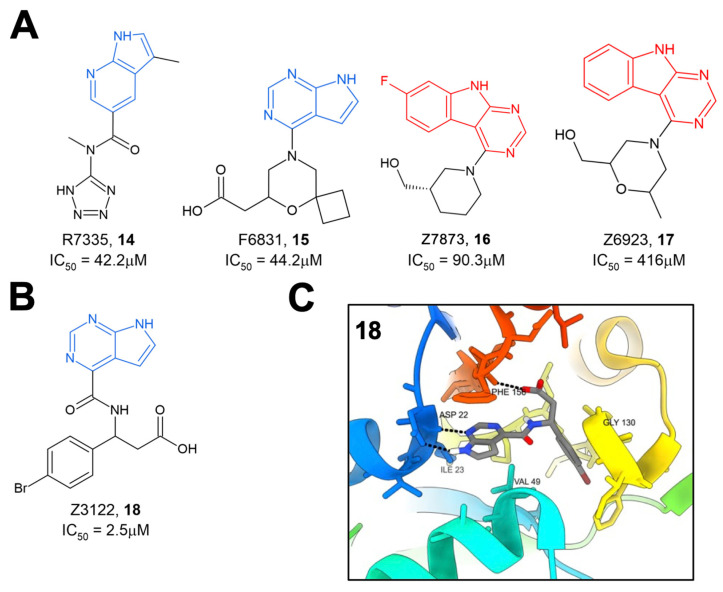
Additional crystallography screening hit compounds. (**A**) Binding to Mac1 by compounds **14**–**17** is facilitated by either pyrrolopyrimidine (blue) or tricyclic pyrimidoindole (red) head group. (**B**,**C**) The structure of the top compound from this crystallography screen, **18** (**B**), and its binding to Mac1 (PDB 5SS9) (**C**) [28].

The same group later performed another molecular docking screen followed by crystallography screens of the top hits [28]. After docking approximately 400 million compounds, 124 were selected for further testing via crystallography and HTRF. Of these 124 compounds, many either bound to Mac1 via crystallography (47 compounds) or demonstrated the inhibition of binding in the HTRF assay with IC_50_ values ranging from 42 to 504 μM (**14**–**17**) (Figure 5A). These hits consistently targeted the adenosine binding pocket, with those compounds that showed activity in the HTRF assay typically consisting of a pyrrolopyrimidine or pyrrolopyridine head group (in blue, Figure 5A) that bound in the adenine binding pocket and stack with F156 and used acidic moieties to interact with the oxyanion subsite, similar to the results obtained from their fragment screen. However, two compounds, Z7873 (**16**) and Z6923 (**17**), instead utilized a tricyclic pyrimidoindole scaffold (in red, Figure 5A) that is closely related to the pyrrolopyrimidine head group. This series was the starting point for optimization towards low-micromolar Mac1 inhibitors by this same group, as outlined below. The top compound from these screening efforts was Z3122 (**18**), with an IC_50_ of 2.5 μM (Figure 5B). This molecule was identified by screening for molecules that docked in the “open” state of the phosphate-binding region, though via crystallography it was shown to bind in the “closed” state. This molecule uses the commonly identified pyrrolopyrimidine to interact with the adenine binding site, a carboxylic acid to make contact in the oxyanion subsite, but notably has a 4-bromobenzyl ring that extends further into the phosphate-binding region than other compounds (Figure 5C).

## 3. High-Throughput Screening Using Both Binding and Enzyme Assays Have Identified Several Mac1 Inhibitors

**Figure 6:**
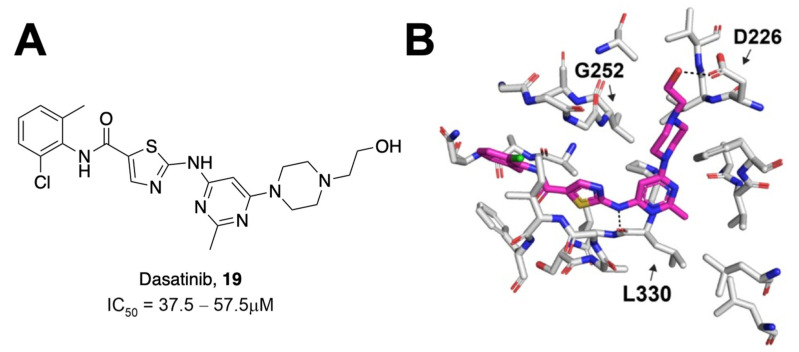
Dasatinib was identified as a Mac1 inhibitor using the ADPr-Glo assay. (**A**) Chemical structure of dasatinib and its measured IC_50_ value. (**B**) Molecular modeling of dasatinib into the Mac1-binding pocket [29].

Most assays that have been used to identify Mac1 inhibitors are based on inhibiting Mac1 binding to ADP-ribosylated peptides. However, a significant aspect of its function is its ability to remove ADP-ribose from proteins through hydrolysis. Dasovich et al. developed a novel technique for the evaluation of ADP-ribosylhydrolase activity, the luminescence-based ADPR-Glo assay, to serve as a high-throughput alternative to gel-based measures of Mac1 enzyme activity and inhibition [29]. This approach utilizes co-incubation of an ADP-ribosylated substrate, Mac1, and the NudF phosphodiesterase. Hydrolyzed ADP-ribose is cleaved by NudF into AMP, the levels of which can be quantified as luminescence with the AMP-Glo kit. Dasovich et al. applied this technique to over 3000 compounds from the Selleck-FDA and LOPAC libraries and identified dasatinib (**19**) as a compound with the specific, dose-dependent inhibition of Mac1 [29]. This compound had an IC_50_ between 37.5 and 57.5 μM (Figure 6A). Surface plasmon resonance analysis confirmed specificity for SARS-CoV-2 Mac1 over the human MacroD2, and molecular docking revealed that only 15 out of 25 contacting residues were conserved between the two enzymes, potentially explaining the selectivity. Dasatinib extended into multiple pockets within the active site of Mac1 (Figure 6B), but biochemical data indicate that it will require further optimization to be a potent Mac1 inhibitor.

**Figure 7:**
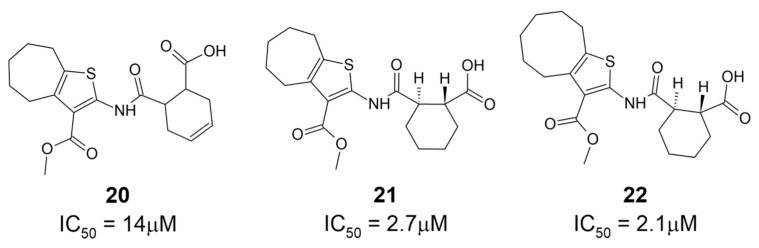
Mac1 binding compounds featuring fused aliphatic and thiophene ring structures [31].

A novel high-throughput ADP-ribose binding assay developed by Sowa et al. is based on the known cysteine ADP-ribosylation of the α_i_ subunits of heterotrimeric G proteins (Gα_i_) via pertussis toxin (PtxS1) [30]. Because the ADP-ribose is attached to a cysteine, it is unlikely to be hydrolyzed by macrodomains, which are only known to hydrolyze ADP-ribose from proteins at acidic residues. In this assay, a YFP-fused Gα_i_ subunit is co-incubated with a CFP-fused ADP-ribose binding protein of interest. Close proximity between the YFP and CFP fluorophores produces a fluorescence energy transfer (FRET) signal that correlates with the substrate binding affinity. Applying this technique, Wazir et al. identified three derivatives of a 2-amine-3-methylester thiophene scaffold with high affinity and specificity for SARS-CoV-2 Mac1 [31]. The initial top hit, **20**, contains a seven-membered aliphatic ring fused with a heteroaromatic thiophene ring and a cyclohexenyl group fused to a central amine linkage (Figure 7). This compound had an IC_50_ of 14 μM for SARS-CoV-2 Mac1 and showed greater levels of inhibition of Mac1 at 100 μM (~80% inhibition at 100 μM) than alternative binding partners PARP9 MD1 (~70% inhibition at 100 μM) or ALC1 (~60% inhibition at 100 μM). The crystal structures of this compound complexed with SARS-CoV-2 Mac1 show that the fused aliphatic and thiophene ring structure forms hydrophobic interactions with F156, while the carbonyl oxygen of the methyl ester group forms hydrogen bonds with the I23 backbone. Iterating on the basic structure of **20** led to the development of derivative **21**, which substitutes the cyclohexenyl unit from **20** with a saturated cyclohexanyl unit in an (*R*,*R*)*-trans* configuration. Further iteration on this structure produced **22**, which substitutes an eight- rather than a seven-membered aliphatic ring fused to the thiophene ring. Both **21** and **22** show improvements in IC_50_ (2.7 μM and 2.1 μM, respectively). Crystal structures of **22** complexed with SARS-CoV-2 Mac1 reveal similar interactions as in **20**, except for a substituted hydrogen bonding interaction between the carboxylate and I32 [31]. Notably, hydrophobic interactions with F156 are enhanced due to the larger eight-membered aliphatic ring and a slight change in F156 orientation. This compound demonstrated improvements in selectivity against ALC1 and also inhibited macrodomains from SARS-CoV (64% inhibition at 100 μM) and MERS-CoV (43% inhibition at 100 μM), suggesting the suitability of this scaffold as a selective inhibitor of viral macrodomains.

**Figure 8:**
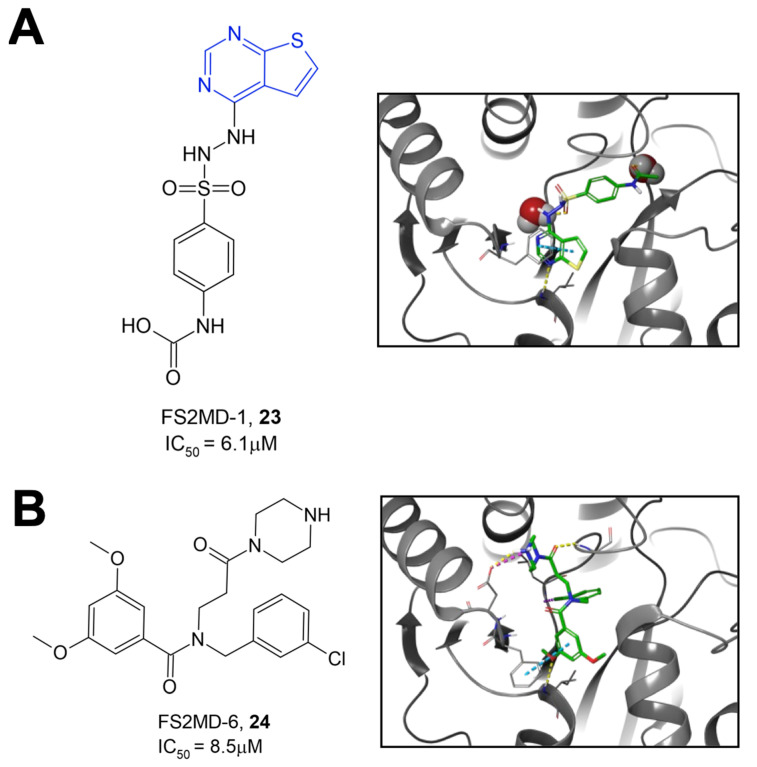
Top hit compounds were identified via high-throughput screening using an AlphaScreen assay. (**A**) Compound **23** contains a thienopyrimidine head group (blue) resembling the pyrrolopyrimidine head groups in compounds identified by Schuller et al. (**B**) Compound **24** contains a *β*-alanine core, a chlorobenzyl group, a methoxy benzoyl group, and a piperazine amide [32].

Roy et al. utilized a previously published AlphaScreen assay and, as an orthogonal assay, a novel fluorescence polarization (FP) assay to screen ~38,000 compounds from the Analyticon, 3D Biodiversity, and Peptidomimetics libraries [32]. Following HTS, the selected compounds were further tested for Mac1 binding affinity using thermal shift assays and for their inhibition of hydrolysis activity using the ADP-Glo assay developed by Dasovich et al. [29]. Of those screened, the highest-performing compounds included compounds FS2MD-1 (**23**) and FS2MD-6 (**24**) with IC_50_ values of 6.1 and 8.5 μM, respectively (Figure 8). Compound **23** contained thienopyrimidine and resembled previously identified compounds from Ekblad and Schuller [26,27]. Compound **24**, which is comprised of a *β*-alanine core, an N-chlorobenzyl group, a methoxy benzoyl group, and a piperazine amide, was the highest-performing compound in the study. In addition to its low IC_50_, the compound demonstrated highest ΔT_m_ values at 1.67 ± 0.21 °C and showed substantial enzyme inhibition in both the ADP-Glo and gel-based assays.

**Figure 9:**
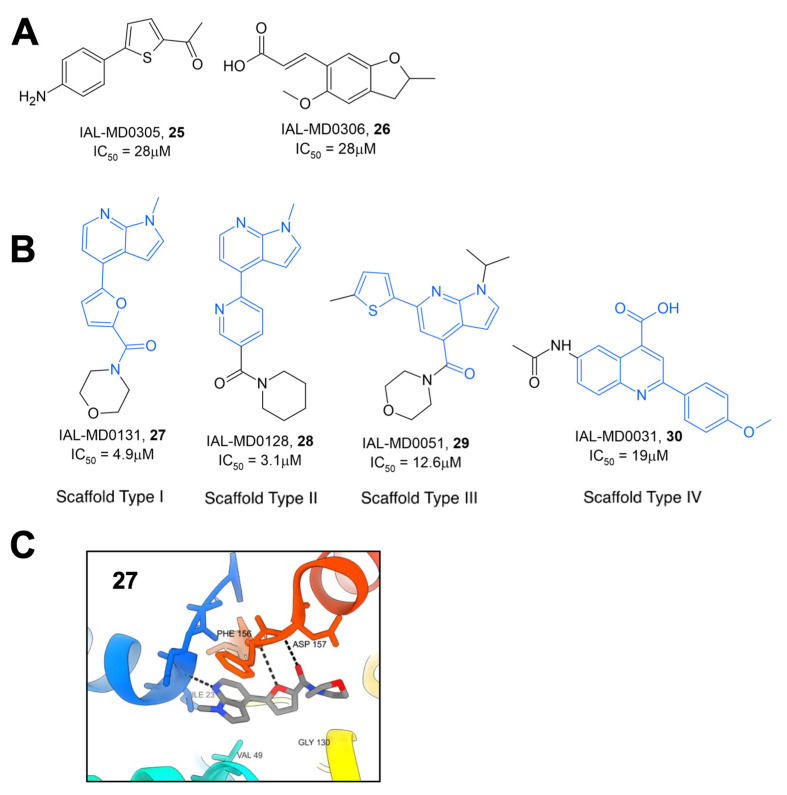
Mac1 inhibitors identified through HTS with the HTRF assay. (**A**) Hits from the BioAscent library. (**B**) Example small-molecule hits generated from the Manchester Institute Diversity Set with primary scaffold types highlighted in blue. (**C**) Crystal structure of **27** within the Mac1-binding pocket (PDB 8C19) [33].

Schuller et al. utilized both virtual and high-throughput screening using the HTRF assay described previously to identify novel Mac1 inhibitors [33]. The virtual screening of 125,000 compounds from the BioAscent library in addition to HTRF screening of 1786 compounds identified two molecules, IAL-MD0305 (**25**) and IAL-MD0306 (**26**), with IC_50′_s of 28 and 18 μM, respectively (Figure 9A). Next, the screening of 10,100 small molecules from the Manchester Institute Diversity Set (MIDAS), as well as an array of FDA-approved compounds, identified four primary scaffold types, three of which contained a pyrrolopyridine core (Figure 9B). Preliminary SAR was able to be gathered due to the availability of several derivatives from each of these scaffold types. Scaffold type I, characterized by a furanyl-pyrrolo[2,3-*b*]pyridine structure, yielded six compounds with IC_50_ values below 9 μM and one at 25.3 μM. The top compound from this set, IAL-MD0131, **27**, contains a morpholine amide and had an IC_50_ of 4.9 μM. Co-crystallization of **27** with SARS-CoV-2 Mac1 showed ligand binding at the adenosine binding site of the ADP-ribose binding pocket (Figure 9C). This was mediated via interactions between the scaffold backbone and the residues I23 and F156, resembling those established by ADP-ribose. Scaffold type II was characterized by a pyridinyl-pyrrolo[2,3-*b*]pyridine, with the top-performing compound from this scaffold being IAL-MD0128, **28**, with an IC_50_ of 3.1 μM. Scaffold III was characterized by a thiophenyl-pyrazolo[3,4-b]pyridine core with the most potent compound identified also containing a morpholino amide IAL-MD0051, **29**, with an IC_50_ of 13 μM. Scaffold type IV was defined by phenylquinoline-4-carboxylic acid, and the most potent derivative identified in this series was IAL-MD0031, **30**, with an IC_50_ of 19 μM.

**Figure 10:**
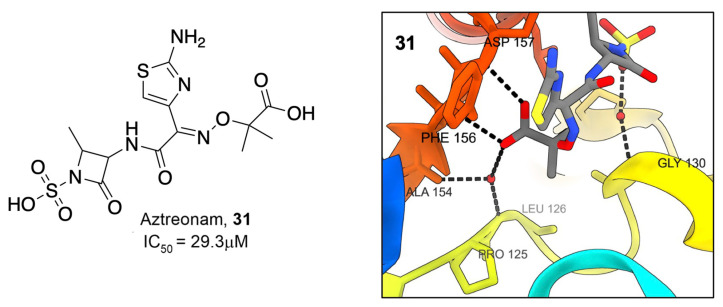
Beta-lactam antibiotic Aztreonam binds Mac1 [33].

Additionally, an in vitro screen of FDA-approved compounds revealed several antibiotics with inhibitory activity against SARS-CoV-2 Mac1. Aztreonam, **31**, a monocyclic beta-lactam, demonstrated an IC_50_ of 29.3 μM and was similarly co-crystallized with SARS-CoV-2 nsp3 (Figure 10). These structures revealed that, rather than binding within the ADP-ribose binding pocket, the compound occupies an adjacent groove coordinated by hydrogen bonding of the carboxylic acid group to the oxyanion subsite of Mac1 by coordinating over a water molecule to the backbone amides of A154 and P125. This observation, combined with the frequency of carboxylic acid groups in a number of already identified Mac1 inhibitors, caused researchers to speculate that this functional group may play similar roles in other compounds. Interestingly, both **27** from the MIDAS library and **31** show π-π stacking with F156 similar to ADP-ribose and many other compounds identified by multiple groups.

## 4. Early Efforts in the Optimization of Fragment Hits to Potent Mac1 Inhibitors In Vitro

**Figure 11:**
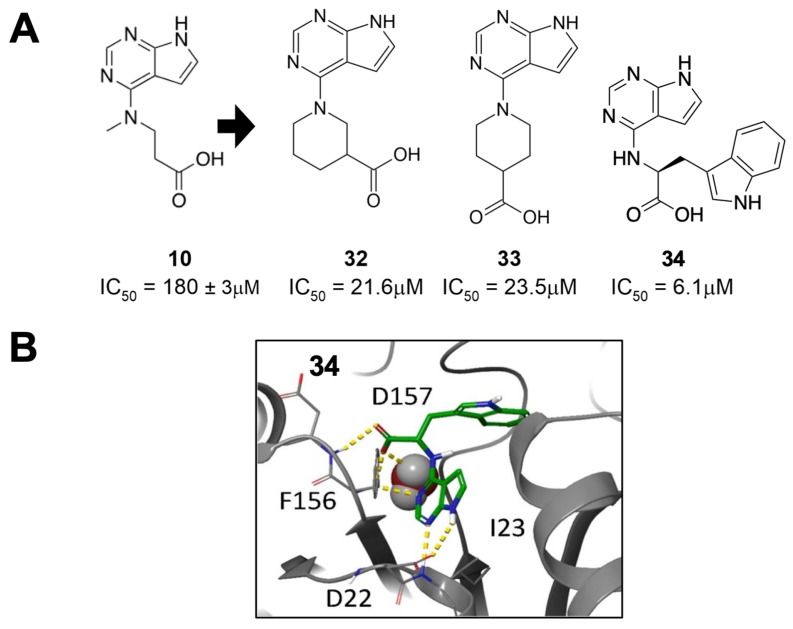
Primary and secondary amino acid derivatives of **10** showed improved Mac1 binding. (**A**) Compounds **32**–**34** improve the IC_50_ of **10** 20- to 30-fold. (**B**) Molecular model of **34** with Mac1 demonstrates that the tryptophan extends deep into the phosphate-binding pocket [34].

Sherrill et al. synthesized and assayed primary and secondary amino acid derivatives that served as iterations on the high-performing 7H-pyrrolo[2,3-d]pyrimidine scaffolds previously identified by Schuller et al. [26,34]. From this optimization, the three highest-performing amino acid derivatives—two secondary and one primary—were demonstrated to have IC_50_ values less than 24 μM (Figure 11)**,** a nearly 10-fold improvement over the original fragments. The two secondary amino acid derivatives, **32** and **33**, incorporated piperidine rings with a carboxylic acid attached at the three- or four-position. As expected, molecular modeling revealed a common feature of hydrogen bonding between the pyrrolopyrimidine core and the D22 and I23 residues of Mac1, while the carboxylic acid moiety from the six-membered piperidine makes hydrogen bonds with the oxyanion subsite. Several primary amino acid derivates also demonstrated significant Mac1 inhibitory activity, including valinate and *β*-amino acid derivates. Of the primary amino acid derivates, the addition of tryptophanate was the most potent, resulting in compound **34**, which had an IC_50_ of 6.1 μM. This result was confirmed in a FRET-based assay, which also showed that **33** had a high level of specificity for coronavirus macrodomains. Finally, the differential scanning fluorimetry of **34** resulted in thermal shift values of >4 °C, which was within 0.2 °C of ADP-ribose. Molecular modeling indicates that the tryptophan moiety extends deep into the phosphate-binding pocket, likely accounting for the increased potency (Figure 11). Nearly all ester derivatives of each tested compound were less potent inhibitors of Mac1-ADP-ribose binding when compared to their carboxylic acid counterparts, suggesting that the carboxylic acid group is important to efficient Mac1 binding. However, several methyl- and ethyl-esters had only mildly reduced activity, indicating the feasibility of creating neutral Mac1 inhibitors using this backbone.

**Figure 12:**
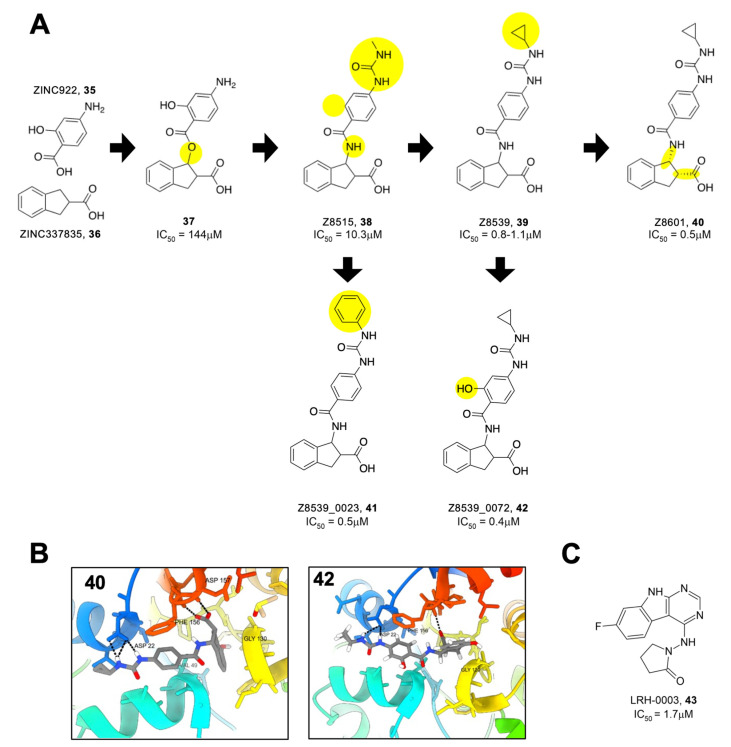
Compounds identified via computational fragment-linking. (**A**) Progressive modifications of **37** led to compounds with sub-μM IC_50_ values (modifications highlighted in yellow). (**B**) Structures of highly potent compounds **40** and **42** (PDB 5SQJ, 5SQW). (**C**) Fragment-linking identified neutral compound with potent inhibitor activity [28].

Gahbauer et al. utilized fragment merging to create the most potent Mac1 inhibitors yet to date. Using an automated fragment-linking approach, termed Fragmenstein, they virtually merged fragment hits and modeled them into the Mac1-binding pocket [28]. These merged molecules were then used as templates to search for available chemical matter in the Enamine REAL database. They ultimately tested 13 molecules for interactions with Mac1 via crystallography and thermal shift analysis. Eight of these hits bound to Mac1 via crystallography, while seven of them induced a thermal shift. Additional analogs of the merger between ZINC922 (**35**) and ZINC337835 (**36**) were purchased, allowing for some initial SAR (Figure 12A). The most potent molecules from these mergers were Z8515 (**38**) and Z8539 (**39**), with IC_50_ values of 10.3 and 1.1 μM, respectively. These compounds share a phenylurea group that stacks with F156 but differ in that Z8539 has a cyclopropyl group that extends further into the adenine binding pocket. Furthermore, upon chiral separation, it was found that the (*R*,*R*) steroisomer, Z8601 (**40**), had an even better IC_50_ value of 0.5 μM. The improved efficacy was explained through crystallography, showing that the indane group of the (*R*,*R*) stereoisomer flipped into the phosphate-binding domain and hydrogen bonds with the backbone oxygen of L126 (Figure 11B). Importantly, these highly potent inhibitors had minimal to no effect against human macrodomains. Next, further modifications to these fragments were created at the cyclopropyl moiety, the phenylurea, and the acid-carrying indane group. From here, several compounds were created with IC_50_ values ranging from 0.4 to 83.8 μM, including at least five with sub-micromolar IC_50′_s. Z8539_0023 (**41**), where the cyclopropyl group was replaced by a phenyl group (IC_50._ = 0.5 μM, thermal shift of 9 °C), and Z8539_0072 (**42**), where an alcohol group was added to the central benzene (IC_50_ = 0.4 μM) (Figure 12B), along with **40**, which are the most potent SARS-CoV-2 Mac1 inhibitors derived to date.

As mentioned above, many of the most potent inhibitors use carboxylic acids to interact with the oxyanion subsite. To address this issue, Gahbauer et al. identified analogs linking the pyrrolopyrimidine or pyrimidoindole to small functional moieties containing hydrogen bond donors or acceptors (e.g., sulfones, hydroxyls, pyridines, or ketones) [28]. Out of 124 possible compounds, 20 were ultimately tested, and 14 were found to bind to the SARS-CoV-2 Mac1 protein via crystallography and 4 via HTRF. The most promising molecule, LRH-003 (**43**), had an IC_50_ of 1.7 μM, not far from the most potent acidic compounds (Figure 12C).

## 5. Discussion/Conclusions

The advancements in applied modeling and biochemical techniques have greatly expanded the repertoire of candidate SARS-CoV-2 Mac1 inhibitors within the last few years. There remain, however, significant challenges which must be addressed before this objective can be fully realized. The presence of acidic side chains in several of the identified compounds has raised potential concerns regarding membrane permeability. However, the potency of several neutral modifications indicates that developing potent cell permeable inhibitors is highly feasible [28,34].

Another major challenge will be to move inhibitor testing from in vitro assays to demonstrating efficacy in cell-based activity and virus replication assays. Russo et al. recently developed an immunofluorescent-based assay for testing inhibitor efficacy in cell culture [23]. This assay is based on the observation that MARylation is drastically increased in cells following polyI:C or IFN-γ treatment. This assay, and potentially others, should allow for cell-based testing, though the dynamic range of these assays may not be very large. Alhammad et al. recently demonstrated that a recombinant SARS-CoV-2 bearing a full Mac1 deletion had only minor defects in virus replication, unless cells were pretreated with IFN-γ, though the virus was extremely attenuated in mice [11]. These results indicate that testing inhibitors for SARS-CoV-2 inhibition in cell culture will be difficult. However, MHV and MERS-CoV Mac1-deleted viruses were unrecoverable, indicating that Mac1 is critical for their ability to replicate. Thus MHV/MERS-CoV may be excellent viruses to test Mac1 inhibitors. In fact, Wazir et al. recently showed that **22** was not toxic to cells and inhibited MHV replication in a dose-dependent manner at mid–high micromolar concentrations, as might be expected considering it is unknown what the IC_50_ of **22** would be for MHV Mac1. Identifying Mac1 targets and biomarkers during infection could substantially aid in demonstrating inhibitor efficacy, making the pivot from in vitro to in situ testing a critical next step for identified compounds.

However, while the SARS-CoV-2 Mac1 has a highly conserved structure and function, variations between coronavirus macrodomains demonstrate the difficulty of applying identified compounds as broad-spectrum antivirals. Commonly identified features such as π-π stacking between F156 and heterocycles, such as pyrrolopyrimidine groups or hydrogen bonding to D22, feature in some of the strongest candidates. However, both SARS- and MERS-CoV encode asparagine in place of F156 [7]. The reliance of many compounds on interacting with the F156 residue creates the possibility for a resistant mutant to arise, though it is currently unclear how such a mutation would affect overall SARS-CoV-2 fitness. This illustrates the inherent difficulty in designing drugs that can inhibit multiple viral macrodomains, as many within the same family contain slight variations in their overall binding scheme that undercut the potency of the established inhibitors. In cases where SARS-CoV-2 Mac1 inhibitors were tested against related macrodomains from SARS- and MERS-CoV, they demonstrated some activity, but it was generally much less than their activity against the SARS-CoV-2 Mac1 [19,31,32,34]. However, as inhibitor development becomes more advanced, there should be opportunities to develop more broad-spectrum inhibitors, or at least SAR should indicate minor changes that could quickly change the specificity of a particular compound from one macrodomain to another.

Despite these challenges, the future of macrodomain inhibitor development is bright, as more compounds with low-to-sub-micromolar activity continue to be identified. With several groups currently working to develop Mac1 inhibitors, it is likely that there will soon be compounds with mid- to even low-nanomolar affinity activity and have appropriate ADME and PK properties for cell and animal testing. With any luck, these efforts will soon fully unlock the potential of utilizing Mac1 inhibitors as research tools and even antiviral therapeutics.
